# P-1102. Educating patient caregivers on hand hygiene: A key step in reducing health care-associated infections

**DOI:** 10.1093/ofid/ofaf695.1297

**Published:** 2026-01-11

**Authors:** Md Shariful Amin Sumon, Golam Dostogir Harun

**Affiliations:** icddr,b, Dhaka, Dhaka, Bangladesh; icddr,b, Dhaka, Dhaka, Bangladesh

## Abstract

**Background:**

Inadequate hygiene compliance among patients’ caregivers could significantly increases the burden of healthcare-associated infections. Strengthening caregivers’ understanding of hygiene management is essential for their active participation in reducing transmission risks during patient care. This study examined the efficacy of an infection control intervention to improve hand hygiene (HH) practices among caregivers.

**Figure:**
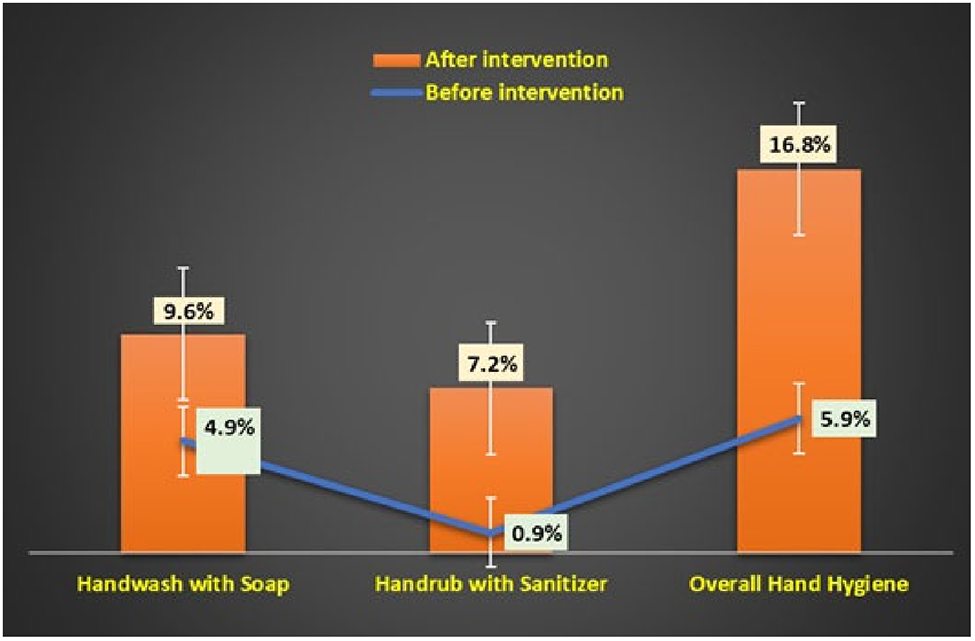
Changes in caregivers’ hand hygiene compliance Impact of Hand Hygiene Intervention among patient's caregivers

**Methods:**

From July to September 2024, we conducted a pre-post-intervention evaluation at four tertiary hospitals in Bangladesh. The intervention package comprised informative messages regarding the use of sanitizer and soap for HH, disposal of medical wastes, management of patient files, and visiting policies. A semi-structured questionnaire was used to collect data from caregivers of admitted patients. Prior to and following the intervention, a two-hour unobtrusive observation was carried out to document the caregivers’ activities and HH practices during patient care. Intervention efficacy was assessed using descriptive statistics and prevalence differences (PD).

**Results:**

A total of 7,152 activities were documented from 234 sets of pre- and post-observation sessions, of which 85.3% required hand hygiene. The intervention resulted in a significant improvement in HH practices among caregivers, increasing from 5.9 to 16.8% following the intervention, with an overall PD of 10.9% [95%CI: 8.9, 14.7; p:< 0.001]. Caregivers were observed to be more adhere to hygiene practices in appropriate waste management [PD: 11.3%, 95%CI: 7.8, 16.7] and proper handling of patient files [PD: 8.4%, 95%CI: 6.9, 12.4]. The intervention led to a notable increase in the self-purchase of sanitizer bottles, rising from 7.7% to 11.2%. However, caregivers-visitors ratio per patient decreased from 3.23 to 1.41 following the intervention.

**Conclusion:**

The infection control intervention led to a significant improvement in caregivers’ hygiene compliance, demonstrating its effectiveness in enhancing infection prevention during patient care. These findings underscore the importance of implementing targeted, caregiver-focused infection control strategies to strengthen patient safety and reduce the risk of healthcare-associated infections within hospital settings.

**Disclosures:**

All Authors: No reported disclosures

